# Mental Health Recovery Process Through Art: An Exploratory Mixed-Methods Multi-Center Study of an Art-Based Community Project

**DOI:** 10.3390/healthcare13101103

**Published:** 2025-05-09

**Authors:** Jaume Cases-Cunillera, Ruben del Río Sáez, Josep Manel Santos-López, Salvador Simó-Algado

**Affiliations:** 1 Research Group on Innovation in Mental Health and Social Wellbeing (ISAMBES), 08500 Vic, Spain; 2 Faculty of Health Sciences and Welfare, Centre for Health and Social Care Research (CESS), University of Vic-Central University of Catalonia (UVIC-UCC), C/de la Sagrada Família 7, 08500 Vic, Spain; 3 Institute for Research and Innovation in Life Sciences and Health in Central Catalonia (IRIS-CC), Ctra. de Roda 70, 08500 Vic, Spain; 4 Osonament-Fundació Centre Mèdic Psicopedagògic d’Osona, C/Josep Maria Selva 2, 08500 Vic, Spain; 5 University Hospital of Vic, C/de Francesc Pla el Vigatà 1, 08500 Vic, Spain

**Keywords:** mental health, art based, recovery, stigma, Photovoice, community, social, connecting and belonging, person centered, mixed method

## Abstract

**Background/Objectives:** Art-based community projects positively impact mental health recovery by fostering creativity, self-expression, and social engagement. Despite growing evidence on participatory art interventions, limited studies have used a mixed-methods approach to examine their effects. The present study examines how participation in the Artistic Couples project influences individuals’ subjective perceptions of recovery, psychological well-being, and self-stigma. **Methods:** This exploratory multi-center study employed an embedded mixed-methods design, integrating qualitative Photovoice methodology with a quantitative pre–post survey. Participants (N = 30) from five mental health institutions across Catalonia engaged in collaborative art creation with local artists. Qualitative data from Photovoice discussions and semi-structured interviews were analyzed using thematic analysis, while quantitative data from standardized measures were examined using paired t-tests and correlation analysis. **Results:** Qualitative findings revealed the following three key themes: (1) artmaking as an artistic couple, emphasizing the collaborative process and art as a means of self-expression; (2) social connections, highlighting increased belonging, emotional support, and reduced loneliness; and (3) understanding mental health recovery, showcasing art’s role in identity reconstruction and personal growth. Quantitative results indicated a significant improvement in the “Connecting and Belonging” subscale of the RAS-DS (t = −2.51; *p* = 0.023), particularly among women (t = −2.85; *p* = 0.019), suggesting enhanced social integration. However, no statistically significant changes were observed in overall recovery, well-being, or self-stigma scores. **Conclusions:** This study provides evidence that participatory community art projects enhance social connections and self-expression, which are key elements of mental health recovery. The findings suggest that creative collaborations facilitate emotional processing and challenge stigma. The improvement in social belonging supports integrating arts-based interventions in recovery-oriented care. Future research should examine long-term effects and gender-sensitive approaches.

## 1. Introduction

Art-based community projects support a positive effect between art participation and mental health improvement [1,2,3,4], offering valuable opportunities to foster creativity and self-expression [5,6]. These practices enable people to engage in their social environment [7], thereby developing stronger social connections [8,9]. Such relationships can significantly reduce the stigma associated with mental health challenges [4,10].

Influenced by consumers and caregivers, the concept of personal recovery in mental health has evolved into a holistic, person-centered approach that incorporates lived experiences and non-medical perspectives [11,12], prioritizing individual experiences over standardized treatments [13,14]. Research suggests that traditional models may not fully address recovery needs or social dimensions [15,16]. The CHIME framework [17] outlines essential elements of recovery—Connectedness, Hope, Identity, Meaning, and Empowerment—derived from a narrative synthesis and a systematic review. These components are validated in research as being central to both the practical application and theoretical understanding of recovery [18,19,20]. Emerging recommendations suggest that recovery-oriented mental health services should adopt person-centered approaches that align with users’ lived experiences [21,22], thus giving the recovery model a more subjective perspective and moving away from professionally dominated interventions [23,24].

Studies indicate that mental health recovery can be enhanced and perceived as a community effort [25]. The collaborative exploration of recovery through creative engagement, as a relational component, presents opportunities to nurture equitable, appreciative, and interconnected communities [26,27]. The body of evidence demonstrating the beneficial effects of creative arts on health continues to grow steadily [28,29]. Notably, community arts initiatives have been linked to promoting self-expression, enhancing skills and achievements, reducing social isolation, and improving self-efficacy [30]. Community-based arts programs provide significant mental health benefits, but their effectiveness can be constrained by challenges related to accessibility and long-term sustainability, particularly in rural or underserved communities [3]. These initiatives promote inclusivity and enhance social connections [8], yet their success relies on the presence of safe, non-stigmatizing environments and consistent opportunities for participation. The authors recommend that future studies should adopt prospective designs that utilize mixed-methods approaches, incorporating validated recovery outcome measures alongside subjective assessments of participants’ perceptions of their recovery process.

Building on these recommendations, the present study aims to examine how a community art project influences participants’ subjective perceptions of their recovery process, psychological well-being, and self-stigma. To the best of our knowledge, no mixed-methods study has yet evaluated the experiences and self-perception of individuals participating in an art-based community project through a participatory research approach. To address the need for a deeper understanding of art-based interventions within the recovery framework, this study focuses on participants from the Artistic Couples project. The purpose is to identify the specific elements of these interventions that provide the greatest benefits to individuals, recognizing that the impact may vary among different participants.

### 1.1. The Study

#### 1.1.1. Aim

The aims of this study are as follows:

(a) To examine the impact of participating in the Artistic Couples project and its influence on subjective perceptions of the recovery process in mental health, psychological well-being, and self-stigma.
(b) To explore the experiences, satisfaction and opportunities for participation in the Artistic Couples project among individuals linked to mental health services.
(c) To understand how mental health recovery is perceived and connected to the experiences within the Artistic Couples project.

#### 1.1.2. About the Artistic Couples Project

The Artistic Couples (translated from the original Catalan name “Parelles Artístiques”) [31] project was launched in 2006 at Osonament, which is a non-profit organization dedicated to promoting the holistic development of individuals with mental health diagnoses and enhancing their quality of life. The project involves the creation and subsequent exhibition of works produced by pairs consisting of an individual with mental health concerns and a local artist. Meetings between participants take place at community services such as artistic workshops, civic centers, urban areas, academic institutions, and art schools. Over seventeen editions, 1089 artists have participated in the program, resulting in 824 artworks across various artistic disciplines including plastic arts, visual arts, performance, and literature. This achievement is largely due to the extensive network of the Artistic Couples project in Catalonia involving diverse mental health institutions that contribute to the initiative. A total of 28 institutions from 15 different counties have participated at least once over the 17 years of the project. Qualitative outcomes from the participants’ process have already been published [32].

## 2. Materials and Methods

### 2.1. Design

This study adopts a qualitatively driven embedded mixed-methods design [33], initiated in October 2022, involving multiple mental health units across Catalonia. Although the initial study protocol included the formation of a control group, the limited number of eligible participants made its creation unfeasible, and only the intervention group could be constituted. The primary focus is on the subjective recovery experiences of participants, explored through the Photovoice method. This process captures the richness and depth of participants’ recovery processes, aligning with the principles of person-centered and recovery-oriented mental health care. Consistent with the subjectivity inherent in the recovery model, this approach highlights not only individuals’ experiences but also the personal significance they attributed to those experiences.

The qualitative methodology is based on the Photovoice method, which uses photography to explore personal experiences and empower participants to document their lives visually [34]. This participatory approach fosters collective meaning through group discussions [35], enabling participants to highlight community strengths, raise awareness of key issues, and advocate for change [36]. Adaptable to diverse populations, Photovoice is particularly effective in mental health contexts [37,38,39], promoting accessibility and emphasizing the principle that “people are the experts of their own lives” ([40] p. 911, [41]). It plays a vital role in recovery by amplifying participants’ voices and encouraging community engagement through dissemination activities like public exhibitions [42].

The qualitative component, anchored in the Photovoice process, is complemented by a quantitative pre–post survey using standardized Patient-Reported Outcome Measures (PROMs) [43,44,45,46,47,48,49,50,51,52], satisfaction surveys, and subjective assessments to provide contextual data, support qualitative data, and triangulate findings.

### 2.2. Recruitment Procedure

The first author contacted the mental health units that were part of the Artistic Couples network and were able to participate in the 17th Artistic Couples project (7 units). The research project and its objectives were presented to each center. Finally, five units agreed to participate. A professional from each institution was in charge of recruitment and was the direct contact with the first author. A total of 40 people from the five units were informed about the research project. Ultimately, 30 participants—7 from Osonament, 7 from Institut Pere Mata, 7 from El Far, 5 from La Muralla, and 4 from Alterarte—agreed to take part in the study. All participants provided written consent. To maintain confidentiality and ensure data protection, participants’ names were anonymized. Participants were included if they were aged 18 or above; participating in the 17th Artistic Couples project; diagnosed with a mental health diagnosis; linked to one of the network institutions; and willing to participate and capable of understanding verbal and written Catalan or Spanish language.

### 2.3. Ethical Considerations

All participants provided written consent prior to their involvement in the study. The first author managed and securely stored the data, with all documentation archived by the institution overseeing the research. To protect confidentiality, participants’ names were anonymized, ensuring compliance with data protection protocols. Participants could withdraw from the study at any time without repercussions. The use of the Photovoice method emphasized the need to safeguard participants and uphold ethical standards, particularly when employing visual methods that involve personal disclosure. In order to ensure the well-being of participants during the study, specific strategies were implemented to address any potential emotional distress arising from participation in the project. During the research, the presence of mental health professionals allowed for immediate support in case any participant experienced discomfort. Throughout the study, no incidents of significant emotional distress were reported by participants. Nonetheless, these protocols were in place to ensure participant safety and to serve as guidelines for replication in other contexts. The research underscored the importance of creating a safe environment and adhering to ethical guidelines throughout the process.

### 2.4. Data Collection

Data were collected in three phases. At the outset of the project (Phase 1), the first author met with participants at each mental health institution involved. Meetings were held in private rooms, with support from institutional professionals being provided when needed. Each participant received an anonymized participant file that was coded to ensure confidentiality.

Questionnaires were completed in small groups of three to four people. While the questionnaires were self-administered, the first author was present to answer any questions or address doubts. Each initial meeting lasted approximately 60–70 min. Participants completed a questionnaire package consisting of the following: sociodemographic information and PROMs—a questionnaire about the process of recovery (QPR) [43], which is a 15-item psychometric tool validated in Spanish [44]; the Recovery Assessment Scale—Domains and Stages (RAS-DS) [45]—the 38-item Spanish version of the tool [46]; European Quality of Life-5 Dimensions (EQ-5D) [47]—a Spanish adaptation [48]; Ryff Psychological Well-being Scale (PWB) [49]—a 39-item Spanish adaptation [50]; and the Internalized Stigma of Mental Illness Inventory (ISMI) [51]—a 29-item Spanish adaptation [52].

Although support was provided to all participants to facilitate questionnaire completion, several experienced difficulties such as reduced attention span, memory problems, and challenges understanding written questions. The complexity and length of some of the scales may also have contributed to these difficulties, especially for participants with cognitive impairments. In accordance with ethical principles, no additional pressure was exerted to obtain responses, and respecting participants’ autonomy was prioritized. As a result, 13 incomplete or invalid questionnaires were excluded from the quantitative analysis.

At the end of the first meeting, the first author collaborated with participants to schedule the next meeting. At this point, Osonament, Alterarte, and Institut Pere Mata began the Photovoice intervention, each forming a unique group. With El Far and La Muralla, challenges in finding joint meeting spaces arose. To accommodate this, the data collection method was adapted in collaboration with institutional professionals, allowing participants to engage in the qualitative part. The first author conducted individual semi-structured interviews with participants from El Far and two focus groups with La Muralla participants.

The Photovoice intervention (Phase 2) involved a series of meetings guided by the study protocol, during which participants were asked to take photos of their daily lives based on specific themes proposed by the research team. The proposed themes included “recovery process”, “art process”, “stigma”, and “well-being”. These themes were designed to help participants reflect on different aspects of their experiences. Accordingly, the photographs could depict a variety of elements—such as objects, places, situations, or people—that participants associated with each theme. Groups discussed whether identifiable images of people could be included and agreed that such photos could be used with the explicit consent of those depicted. Participants committed to respecting and protecting the data generated in the group.

The first author offered cameras for participants to use, but all chose to use their own smartphones. Before each session, the first author collected and uploaded participants’ photos to a secure computer and printed them for use in discussions. Participants were encouraged to provide titles and short descriptions for their photos if desired. The groups were guided using a modified SHOWED prompt [53], which is a question framework commonly used in participatory methods such as Photovoice that encourages critical reflection through the following steps: What do you see? What is really happening? How does this relate to our lives? Why does this situation exist? How could this image educate others? What can we do about it? Sessions were scheduled collaboratively with participants.

All Photovoice sessions lasted 1–2 h and were conducted in rooms provided by the institutions, equipped with necessary materials (e.g., tables, chairs, blackboards, and artistic supplies). At the end of the meetings, participants completed a satisfaction questionnaire. All sessions were audio-recorded and transcribed verbatim with prior consent from participants. The first author also took detailed notes during the sessions to document observations.

The last phase (Phase 3) encompassed the administration of the same standardized questionnaires from Phase 1. The first author also collected a self-developed survey asking about the satisfaction of participants in their “Artistic Couples” experience. Finally, a self-made patient-reported experience measures (PREM) was assembled. The PREM was designed based on a general review of the relevant literature on participant experiences in community art projects, rather than being directly adapted from a specific existing scale. Its aim was to capture subjective aspects of participants’ experiences related to the intervention. The psychometric properties of this instrument were not formally assessed, as it was intended primarily for descriptive and exploratory purposes. Both the satisfaction survey and measure of experience were anonymized, and no participant codes were linked to responses.

### 2.5. Data Analysis

Qualitative data obtained from the Photovoice process were analyzed using Thematic Analysis following Braun and Clarke’s approach [54]. ATLAS.TI 24 was the software used to analyze qualitative data. Once the data were transcribed, the first author read and re-read the data to become familiar with the information. By identifying text fragments, codes were generated. These codes formed the basis for identifying patterns and themes in the text, allowing for the systematic organization of information. The second and third authors conducted a second coding. Then, a general revision of the generated codes was carried out. Afterwards, codes were grouped into categories, which were then consolidated into broader themes, allowing the researchers to classify and link common categories. Finally, the authors refined and reviewed the emerging themes.

A quantitative analysis was conducted using SPSS 29.0.2.0, incorporating various statistical approaches to explore the data. A descriptive analysis was performed to characterize the variables and provide an overview of the sample. Paired-samples t-tests (or Wilcoxon signed-rank tests, if data were not normally distributed) were used to assess changes in key variables before and after the intervention. Subgroup analyses were conducted using independent-samples t-tests (or Mann–Whitney U tests), and one-way ANOVA was used to compare differences among multiple groups. Finally, correlations between variables were examined using Pearson’s (or Spearman’s) correlation coefficients, as appropriate. The process was reviewed and refined by the co-authors.

## 3. Results

### 3.1. Sociodemographic Data of Participants

A final sample of 30 participants (Table 1) was included in the study. An equal number of women (*n* = 15; range: 18–65 years; mean: 44.13 years; sd = 15.49) and men (*n* = 15; range: 22–59 years; mean: 44.40; sd = 13.21) participated in the study. Mental health diagnoses were established by clinicians at the participating mental health units and recorded in the patients’ medical records. For the purposes of this study, diagnoses were classified according to the DSM-V handbook [55] according to the research team.

### 3.2. Qualitative Findings

Data analysis disclosed multiple and interconnected findings that align with the objectives of the study. The findings are organized into four main themes and subthemes (Table 2). Some narratives are presented without the photo to ensure confidentiality. Additionally, group conversations and complementary experiences were included. A satisfaction questionnaire assessing the Photovoice sessions (Table 3) is also presented.

#### 3.2.1. Artmaking as an Artistic Couple

Creative and collaborative process

**Figure 1:**
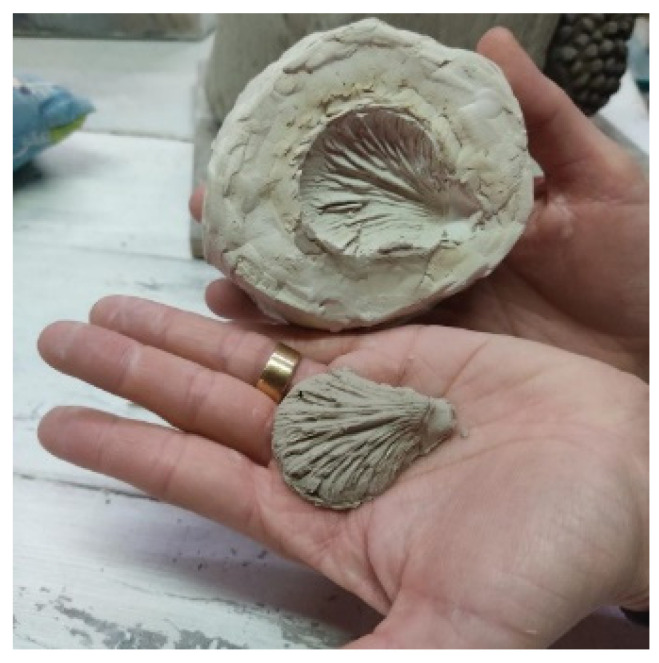
A photo from Participant 4 used to describe her creative process, entitled “The mold”.

Participants emphasized the value of engaging in the creative process over focusing solely on the final product, aligning with participatory art principles. Participant 3 reflected that “Things do not appear; things are created”, highlighting the significance of the journey. Participant 4 described her experience as follows:

“We started walking in the forest, collecting elements like pine cones and logs. From there, we built without a set plan, creating something with volume. I enjoyed the entire process—making the mold, turning it, and documenting it with my artistic partner’s photos” (Figure 1).

Participants found enjoyment in the “making-of” aspect of their work. For example, some participants expressed a preference for the process of creating, enjoying even the “behind-the-scenes” moments—sometimes valuing it more than the final artwork itself. This reflects a broader appreciation of the journey, as well as the therapeutic impact of engaging deeply with the process, capturing moments, and recording the stages of creation, which offered a lasting reminder of their efforts and growth.

The dynamic between clients and local artists also revealed power imbalances, as the artists’ expertise sometimes overshadowed collaborative efforts. Yet, these dynamics highlighted the balance between technical guidance and creative expression.

To express or to explain

Art served as a tool for participants to express emotions and thoughts that are difficult to articulate verbally. Participant 1 noted that “Art expresses what you cannot with words”. Through art, they express not only their feelings but also attempt to make sense of them, thereby facilitating both self-understanding and communication with those around them. Art acts as a mirror of their internal states, enabling others to understand their experiences more accurately.

For Participant 25, art became a channel to release emotions from a difficult work situation.

“My work is called incomprehension. I didn’t know how to capture it, but after brainstorming, I realized it reflected my struggle at work, where colleagues didn’t treat me well. That’s when the idea to capture incomprehension in a painting came to me”.

Art through nature

**Figure 2:**
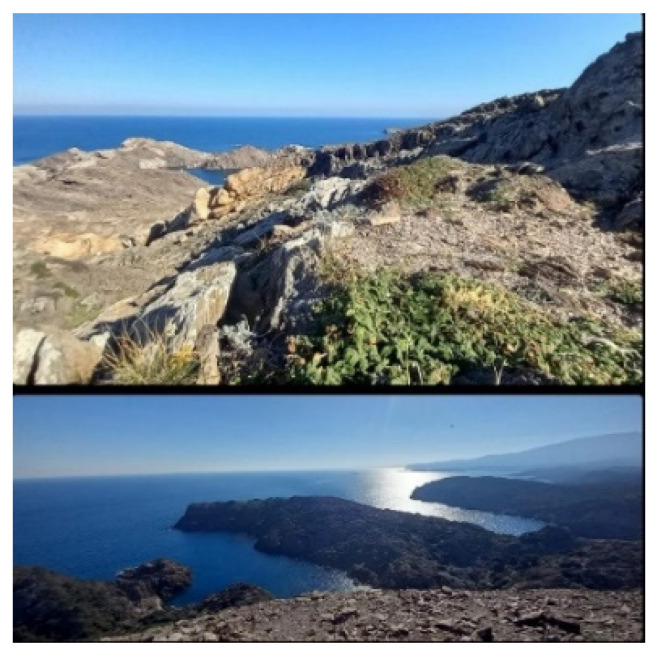
A photo from Participant 1 entitled “Keep your love for nature, because it is the true way to understand art more and more”.

Some participants engaged in the artistic process within a natural setting, which became a significant space for reflection, inspiration, and connection. Participants integrated natural elements into their practices, highlighting the interplay between nature and creativity. During a group discussion, Participant 1 shared a landscape photo alongside Vincent van Gogh’s quote (Figure 2).

This sparked a debate about whether nature is art or merely a canvas for human creativity. While some, like Participant 1, viewed “nature as art”, others, like Participant 2, argued that “art is generated solely by human beings”.

#### 3.2.2. Social Connections

A recurring theme among participants was the dynamic exchange of personal and artistic experiences within the artistic couple process. Some participants delved deeply into personal topics, while others maintained a primary focus on the art itself. These interactions often reflected the level of connection and trust developed between partners.

A conversation starts

Participant 23, the youngest participant at 19 years old, collaborated with two high school students (local artists) to create graffiti artwork. She described how their process began with a two-hour conversation, focusing on personal topics like dreams and emotions. This dialog inspired their project, which explored adolescence through a character navigating emotional worlds such as anger, sadness, fear, euphoria, and shame. Participant 23 explained the following:

“When we started, we first got to know each other and spent two hours just talking. Only talking. We didn’t talk about the artwork; we talked about ourselves. We realized we had things in common. The topic of emotions came up, especially the world of dreams and the subconscious. The main idea was to create a character that went through different emotional worlds, representing adolescence. While we were sketching, I came up with the idea of ‘teenmare’, which is a fusion of ‘teenager’ and ‘nightmare’. We depicted anger, which is a very common emotion among teenagers. Sadness was also included because it’s a time when you begin to realize that life isn’t the world you thought it was when you were a child. Problems start to arise, and you have to decide what to do with your life. Fear was represented because adolescence brings many changes, and we have to try new things and choose our future. Fitting in with others also creates fear. Then there was euphoria and shame, the chains that stop you from being or doing what you want. I had never created an artistic piece with someone else before. I had always worked individually. Relationships are very difficult for me, but with them, it just clicked instantly. It was very easy, and everything went really well. I was nervous in this regard, but I came out delighted”.

Their conversations laid the foundation for trust, collaboration, and creativity, blending personal insights with artistic expression to address the transitional challenges of adolescence.

To share with my artistic couple

Interactions between participants often extended beyond their artistic projects, fostering deeper personal connections. Such exchanges created spaces for trust and understanding. However, they also highlighted challenges. Participant 21 recounted an initial conflict with her partner, who treated her unequally due to her mental health.

“I said, ‘No, we are equal.’ People are not bad; they are ignorant”.

By addressing the comment directly, she fostered mutual respect, concluding that “The process went very well, and now we are friends”. This moment underscores the importance of open communication in relation to challenging stigma and promoting understanding within partnerships.

Solitude

Participants reflected on solitude as both a challenge and an opportunity for growth. While some struggled with being alone, they also found it could foster creativity and self-expression. Several participants reflected on the role of solitude in their recovery, with some struggling to cope with it while others found it a source of creative inspiration. For some, solitude became a companion that fostered artistic expression and personal reflection. However, the Artistic Couples project provided a space to counteract loneliness by fostering social connections through shared creative experiences. Collaborative art making not only strengthened participants’ sense of belonging but also enhanced their mental well-being, offering a meaningful way to engage with others and the artistic community.

#### 3.2.3. Understanding Mental Health Recovery

Mental health recovery is a deeply personal and multifaceted journey that intertwines emotional, social, and creative dimensions. The outcomes of this project reveal how participants navigated their individual paths, using art and meaningful activities as tools for self-expression, empowerment, and connection. Through their experiences, we gain insight into the complexities of recovery, from overcoming stigma and personal struggles to rediscovering identity and finding joy in everyday moments.

Overcoming my current situation

**Figure 3:**
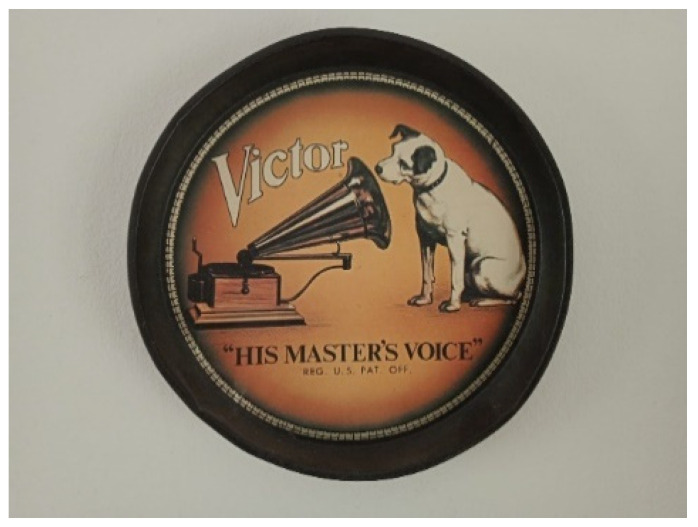
Title: His Master’s Voice.

Participant 2 reflected on his lifelong relationship with music, sharing a photograph of the iconic His Master’s Voice logo, which holds personal significance from his time working at RCA Victor (Figure 3). He explained the following:

“Music was the reason why they stigmatized and damaged my brain. But it’s also always been the center of my life”. As he grew older, personal circumstances distanced him from music. He shared, “I’ve really been a fish out of water for 15 years. That’s why it feels so good to be making music again and to have the whole studio set up. I feel like I am regaining my identity as a musician”. When he prepared the studio, he invited his artistic couple to work there. “And for me, this is a very, very significant aspect of my recovery because what I needed was to regain my artistic identity as a musician”.

Some participants experienced emotional challenges during their creative process. One participant was unable to complete their work due to personal reasons, while others had fewer meetings due to external circumstances.

“Don’t treat me this way”

Participant 22 shared her journey of coping with mental health stigma, using three symbolic photographs to illustrate her experiences. She recounted how her challenges began at 15 years old, with panic attacks, anxiety, and depression. Despite these struggles, she pursued her studies and career by “putting on a mask” to appear normal.

“Even when I was shattered inside, I would dress well, do my hair, put on makeup, and just carry on. Now, I know I have bipolar disorder, but it took me a long time to accept it”. Art became her refuge, stating that “I use art to express my emotions and feelings but also as a way to entertain myself because I enjoy it”. Her second photo, titled “Don’t Treat Me This Way”, depicted a collage of stigmatizing phrases she had encountered, such as “What you want is to avoid working” or “Others have it much worse than you”. In contrast, her third photo, “Get to Know Me First and Discover Who I Am”, revealed empowering statements like: “I’m fun, I want to make a difference” or “Ask me if you don’t understand”.

Reflecting on the Artistic Couples project, she acknowledged the sensitivity of participants but noted occasional moments of discomfort:

“They mean well, but it can still feel bad when they explain things as if you were a child”.

Her narrative highlights the dual role of art as a tool for self-expression and a means of confronting societal stigma, underscoring her resilience and the need for greater understanding.

Engaging in meaningful activities

**Figure 4:**
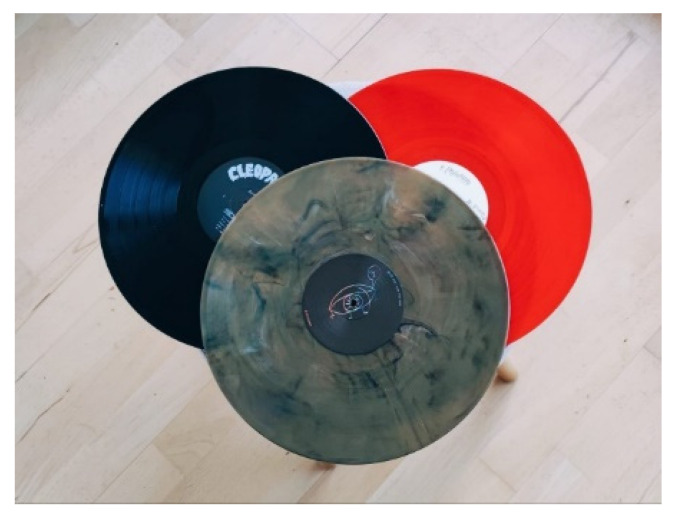
Title: Plasticized emotions.

Participants highlighted the importance of engaging in both artistic and non-artistic activities to support mental health recovery and provide meaningful ways to occupy their time. Examples included the following:

Listening to music: “Music makes me feel powerful emotions with every song I love”, said Participant 3, who chose colorful vinyl records to represent its impact (Figure 4).

Climbing: Participant 1 also described the exhilaration of a via ferrata [protected climbing route found in mountain regions], stating “It’s the adrenaline, feeling alive, and enjoying nature”.

Other activities: Archery, visiting museums, and spending time in nature were also mentioned as meaningful practices.

These activities not only provided enjoyment but also contributed to participants’ emotional well-being and connection to the world around them.

### 3.3. Quantitative Findings

The pre- and post-intervention analysis provide insights into the project’s impact on participants’ mental health recovery, quality of life, and self-stigma (Table 4). Of the 30 participants initially recruited, 17 (56.7%) completed the questionnaires and were included in the quantitative analyses. The mean age of this subsample was 36.9 years (SD = 13.1), ranging from 18 to 58 years. Regarding gender, ten were women (58.8%) and seven were men (41.2%).

In terms of diagnostic categories, eight participants (47.1%) were classified as having psychotic disorders, four (23.5%) as depressive disorders, three (17.6%) as personality disorders, and two (11.8%) as bipolar disorders. With respect to civil status, 12 participants (70.6%) were single, while the remainder were married, divorced, or in a partnership. Most lived with their families (10 participants; 58.8%), while others lived alone (5; 29.4%) or in supported accommodation (2; 11.8%). Educational levels varied—seven participants (41.2%) had completed secondary education, six (35.3%) had higher or university education, three (17.6%) had completed upper secondary education, and one (5.9%) had only primary education. Regarding employment status, only one participant (5.9%) was currently employed, while the majority were unemployed (29.4%) or on temporary or permanent disability (52.9%), with two participants (11.8%) classified as having unspecified or other work status. The remaining 13 participants did not complete the questionnaire due to difficulties in comprehension and concentration, likely related both to cognitive challenges associated with their mental health conditions and to the complexity of some of the scales used. Moreover, a satisfaction questionnaire assessing participants’ experiences in the project (Table 5) and a measure of PREMs (Table 6) were included. It is the latter that highlights the high levels of pleasure and well-being generated by the experience received in the research.

Although overall recovery as measured by the QPR and RAS-DS showed no statistically significant changes, the “Connecting and Belonging” subdomain of the RAS-DS revealed a notable improvement (t = −2.51; *p* = 0.023), suggesting that participants experienced increased social integration and support. The effect size (Cohen’s d) for the paired-samples t-test was 0.44, indicating a moderate effect. The overall recovery as measured by the RAS-DS (Recovery Assessment Scale—Domains and Stages) showed an increase in mean scores from 111.18 (SD = 19.69) pre-intervention to 116.71 (SD = 17.82) post-intervention, with the median rising from 113 to 118. Although the improvement was not statistically significant (t = −1.525; *p* = 0.147), this increase in scores suggests a positive trend in participants’ perceived recovery. Regarding quality of life and psychological well-being, no significant changes were observed in the overall EQ-5D and PWB scores. Lastly, self-stigma levels, as assessed by the ISMI, remained stable across all domains, indicating that while stigma reduction was not significant, the intervention did not exacerbate negative self-perceptions.

**Figure 5:**
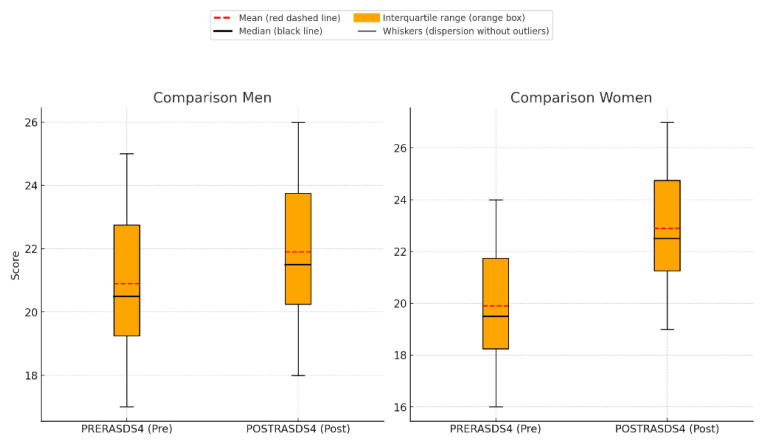
Boxplot comparison of pre- and post-intervention scores (PRERASDS4 and POSTRASDS4) for men and women.

The analysis of the RAS-DS “Connecting and Belonging” subscale revealed significant differences in recovery trajectories based on gender. However, given the small sample size and the imbalance between male and female participants, these results should be interpreted with caution. Women demonstrated a statistically significant improvement, with mean scores increasing from 20.20 (SD = 3.39) pre-intervention to 22.30 (SD = 3.23) post-intervention (t = −2.85; *p* = 0.019) (Figure 5). The effect size (Cohen’s d) for the paired-samples t-test was 0.63, indicating a moderate effect. In contrast, men showed no significant change, with mean scores shifting only slightly from 21.00 (SD = 3.16) to 21.43 (SD = 3.36) (t = −0.55; *p* = 0.604). The direct comparison of improvements between genders yielded no statistically significant differences (t = −1.55; *p* = 0.142), though women exhibited a higher mean change (2.10 for women vs. 0.43 for men). Age-based analyses, including an ANOVA comparing three age subgroups (18–30, 31–50, and 51+), did not show statistically significant differences in recovery scores between groups (F = 0.45, *p* = 0.649 for all participants; F = 1.46, *p* = 0.295 for women only). Notably, older women (51+) showed the highest mean improvement (4.50; SD = 3.54), whereas younger and middle-aged women (18–30 and 31–50) exhibited smaller changes (1.50, SD = 2.08, and 1.50, SD = 1.73, respectively). While these findings were not statistically significant, they suggest that age and gender may influence recovery experiences and warrant further exploration. The results show a low positive correlation between women’s age and improvement in the subscale (r = 0.278), but it is not statistically significant (*p* = 0.436), indicating that there is not enough evidence to confirm a relationship between these variables in this sample.

The one-way ANOVA showed no significant differences in improvement across diagnostic categories (F = 1.264; *p* = 0.327). However, the “Depressive Disorders” group exhibited the highest improvement trend (M = 3.00). The number of participants who completed the questionnaires in each diagnostic category was as follows: “Psychotic Disorders” (*n* = 8), “Bipolar Disorders” (*n* = 2), “Depressive Disorders” (*n* = 4), and “Personality “Disorders” (*n* = 3).

## 4. Discussion

This mixed-methods study explored how the Artistic Couples project influences participants’ mental health recovery, psychological well-being, and self-stigma while also examining their experiences, satisfaction, and the opportunities created through participation. Photovoice emerged as the central methodology, enabling participants to take an active role in the project’s development and offering a platform to express their emotions and insights [56]. Through their photographs and group discussions, participants highlighted key themes, such as artmaking as a collaborative process, social connections, and their journey toward understanding mental health recovery. It has been stablished that mental health recovery concepts are effectively communicated by visual arts-based research [57,58].

Participatory art activities such as the Artistic Couples project encourage individuals to express themselves creatively, promoting autonomy in self-expression [59]. The findings highlight those participants that valued the creative process itself, emphasizing the journey over the final product. This aligns with participatory art principles, where making, experimenting, and collaborating become central to personal and collective growth. Art also functioned as a powerful means of communication, allowing participants to externalize emotions and experiences that were difficult to verbalize [60]. Whether through visual metaphors, symbolic representations, or the physical act of creating, art provided a language of expression beyond words. Art can contribute to reducing the stigma associated with mental health [1,61], as exemplified by Participant 22, who directly confronted stigma with her artistic couple. However, this study did not find statistically significant changes in self-stigma.

Furthermore, nature emerged as a significant element in the artistic process, serving as both inspiration and a reflective space. The integration of natural materials and outdoor settings enriched participants’ creative experiences, reinforcing the interplay between artistic creation and environmental connection. However, this relationship between nature and artistic expression requires further exploration [62], particularly in understanding how natural spaces shape and enhance the self-perception of individuals engaged in creative practices.

Quantitative results revealed that while overall recovery and well-being measures (RAS-DS and PWB) showed trends toward improvement, only the “Connecting and Belonging” subscale of the RAS-DS demonstrated statistically significant changes (t = −2.51; *p* = 0.023), suggesting enhanced social integration and support. Gender-specific analyses showed that women experienced a significant improvement in this domain (t = −2.85; *p* = 0.019), whereas men did not show significant changes. These findings underline the importance of targeted approaches to address gender-specific needs in community-based art interventions. The greater improvement in social connectedness observed among female participants may be related to several factors, such as cultural diversity, socio-economic status, or prevailing gender roles, although we cannot draw firm conclusions from the current data. A review emphasizes that women participate in community art projects in greater numbers than men [63], highlighting the importance of paying particular attention to the gender gap in mental health services, as noted in other studies [28]. However, the small sample size limits the robustness of these quantitative results, which should be interpreted with caution. The qualitative findings clearly demonstrate a positive experience among participants, with narratives highlighting improvements in social connection, self-expression, and emotional well-being. Solitude, a recurring theme, was viewed both as a challenge and an opportunity for introspection and personal growth, which aligned with prior studies suggesting that engaging in social interactions during art activities helps reduce feelings of loneliness [64].

The results of this study align with the CHIME framework [17], which highlights Connectedness, Hope and Optimism, Identity, Meaning, and Empowerment as essential components of mental health recovery. Participants reported emotional release, self-expression, and positive experiences, contributing to a sense of hope and optimism surrounding their recovery journey. The meaningful engagement in artmaking fostered a sense of purpose and value. Moreover, using PROMs allowed us to focus on patient-centered needs, fostering empowerment, and providing a sense of meaning, aligning with a holistic mental health approach [65]. Self-perception emerged as a cornerstone for reclaiming autonomy and reinforcing self-worth, ultimately supporting recovery outcomes.

Satisfaction surveys underscored participants’ positive experiences with the Artistic Couples project, with high ratings for activity functionality (mean = 8.9; SD = 1.53) and overall satisfaction (mean = 9; SD = 1.01). Participants praised the opportunity to share meaningful moments, explore diverse forms of art, and connect with others, despite challenges like scheduling conflicts and occasional mismatches with artistic partners. The project’s ability to create a supportive and inclusive environment was also reflected in the positive feedback regarding the research process (PREMs), with all respondents indicating that they were treated respectfully and found the study easy to understand. However, it should be noted that participants’ feedback in the PREMs reflects their overall perception of the entire research process (including both the qualitative and quantitative components). Therefore, there may appear to be a contradiction, as some participants were unable to take part in the quantitative assessments due to difficulties in completing the questionnaires.

### 4.1. Practical Implications

Art can play a key role in improving mental health, well-being, and social participation [66]. This study highlights the potential for integrating participatory art interventions, such as the Artistic Couples project, into mental health recovery programs to enhance self-expression, social connection, and emotional well-being. Although a significant improvement was observed in women, the small and unbalanced sample size does not allow for definitive conclusions regarding gender differences. Further research with larger and more balanced samples is needed to explore possible gender-specific trajectories in recovery, while the use of natural settings and collaborative art processes suggests additional therapeutic benefits that should also be examined in future studies. Empowering participants by involving them as co-creators, rather than as passive recipients, fosters a sense of ownership and resilience in their recovery journey. In addition, a Photovoice exhibition was held to address the community population. Addressing logistical and power dynamics challenges, such as ensuring equitable relationships between participants and professional artists, can further enhance the program’s effectiveness. A key consideration is the potential for emotional vulnerability and psychological distress when participants take part in the project. Support systems within art partnerships are essential to help navigate emotional journeys safely. This includes the presence of mental health professionals or peer support networks [67,68], where experienced participants provide guidance and assistance.

### 4.2. Study Limitations

This study presents several limitations that should be considered when interpreting the results. The high rate of incomplete questionnaires may have introduced bias into the results, as missing data were likely related to cognitive difficulties or the complexity of the scales. Therefore, the findings should be interpreted with caution, as the final sample may not be representative of the overall population initially recruited. One important limitation of the study is the absence of a control group, which was originally planned in the study protocol. Due to the insufficient number of participants meeting the inclusion criteria, it was not possible to form both an intervention group and a control group. As a result, the ability to attribute observed effects solely to the intervention is limited, and caution should be exercised when interpreting the findings. Additionally, exploring the long-term effects of such participatory art interventions may provide further insights into their potential for the sustained impact on mental health recovery, while also considering diagnostic categories. Second, the sample size was too small to ensure that the statistical significance observed in the quantitative analyses was robust enough to definitively confirm the intervention’s impact. However, the results suggest a trend toward stabilization or even improvement. Third, the findings may be influenced by the specific sociocultural context of the participants, as they were all from a specific geographic region. This may limit the generalizability of the results. Finally, logistical challenges arose as the first author was responsible for leading and supervising all Photovoice sessions and administering questionnaires across various geographic areas. This could have affected the consistency of data collection.

## 5. Conclusions

This study highlights the impact of participatory art interventions in mental health recovery, particularly through the Artistic Couples project. The findings demonstrate that engaging in creative collaboration fosters self-expression, social connectedness, and a renewed sense of identity, aligning with the CHIME framework. Notably, significant improvements in social integration were observed, especially among women, reinforcing the importance of gender-sensitive approaches. While overall recovery and self-stigma measures showed no statistical significance, qualitative insights reveal the deep personal impact of artistic engagement. Addressing power dynamics in collaborations and integrating nature-based elements may further enhance the therapeutic benefits of such initiatives. Despite limitations such as sample size constraints and the lack of a control group, this study underscores the need for sustained, inclusive, and structured art-based programs within mental health services to promote holistic well-being and long-term recovery.

## Figures and Tables

**Table 1:** Study participants (N = 30).

Variables	N	%
Age (years)		
18–30	8	26.66
31–50	10	33.33
51–65	12	40
Gender		
Women	15	50
Men	15	50
Civil status		
Single	22	73.33
Married	3	10
Divorced	2	6.66
Unspecified	2	6.66
Unmarried couple	1	3.33
Living with		
My family	18	60
Alone	7	23.33
Supported household	2	6.66
In a group	2	6.66
With partner	1	3.33
Academic training		
University degree	7	23.33
Secondary education	6	20
Primary education	5	16.66
Advanced education	4	13.33
High school	4	13.33
Without studies	2	6.66
Third-cycle university studies	1	3.33
Unclassified	1	3.33
Job situation		
Transitory/permanent incapacity	12	42.85
Unspecified	8	28.57
Unemployed (with or without subsidy)	4	14.28
Active (working)	3	10.71
Retired	1	3.57
Mental health diagnoses categories (main)		
Schizophrenia spectrum and other psychotic disorders	14	46.67
Bipolar and related disorders	7	23.33
Depressive disorders	4	13.33
Personality disorder	4	13.33
Disruptive, impulse control, and conduct disorders	1	3.33
Institution (Region)		
Osonament (Osona)	7	23.33
Institut Pere Mata (Tarragonès)	7	23.33
El Far (Vallès Oriental)	7	23.33
La Muralla (Tarragonès)	5	16.66
Alterarte (Maresme)	4	13.33
Specific service		
Community rehabilitation service	16	53.33
Social club	14	46.66
Artistic discipline used in the project		
Visual arts	25	83.33
Literary arts	3	10
Music	1	3.33
Applied arts	1	3.33

**Table 2:** Overarching themes, sub-themes, and quotes from participants.

Themes	Sub Themes	Quotes
Artmaking as an artistic couple	Creative and collaborative process	“The final work, the result, is very important because it is what is seen, the visual aspect. But I believe that the meaning, the process, is equally important”—Participant 23
		“It also helps me relax, enjoy the process, and it’s a moment we share—an hour and a half, two hours. And many times, we meet up and don’t even paint; we just go to have a chat”—Participant 1
	To express or to explain	“It wasn’t therapy for me, but a way to explain what happened to me or how I was inside. As a child, I didn’t express myself clearly, and they always said, ‘Get me a drawing’”—Participant 23
		“For me, it’s about understanding myself and communicating what I do not understand about myself. I try to make sense of it and hope others can as well”—Participant 21
		“It helps me a lot to express, to externalize”—Participant 19
	Art through nature	“My process has been in a wonderful place, in the middle of nature. The calm and disconnection that I have had. For me, it has been a very beautiful and wonderful experience”—Participant 4
		“Contact with nature is very healthy for me”—Participant 30
Social connections	A conversation starts	“We talked a bit about what we liked to paint or what we liked to express. I was in a process when Artistic Couples started where I felt overwhelmed. I thought, if I’m going to dedicate so much time to a project, to a piece, working with someone where it doesn’t just depend on me, I want it to be personal. And, in fact, that’s how it was”—Participant 1
	To share with my artistic couple	“I think that when you have feeling and stick more you relax and end up talking about personal things. So, we talked about our things. They had nothing to do with ceramics. His life, my life, this happened to me, it happened to me the other…”—Participant 4
		“So now I really enjoy being in contact with my artistic couple and seeing that she adapts to what I do, and that what she suggests to expand my work also appeals to me. I mean, it’s a very interesting and productive exchange”—Participant 2
	Solitude	“Being an artistic couple helps me get through unwanted loneliness”—Participant 6
		“The power of feeling connected to others… helps me grow. It’s therapy for me”—Participant 8
		“I meet more people, expand my circle of friends, and experiment”—Participant 19
Understanding mental health recovery	Overcoming my current situation	“I tried to paint, but nothing came out. I was struggling and felt like I wasn’t making any progress. It was overwhelming, and I decided I wanted to stop. I felt very anxious”—Participant 24
		“I wanted to approach Artistic Couples in connection with my personal process. Like, how do I feel right now? I feel a bit like ‘hear, see, and stay silent’, and I don’t want to feel that way”.—Participant 1
	“Don’t treat me this way”	“My artistic couple told me, I had prejudices about how you would be, and, you’re just a very normal person’. That whole idea of normality—like, you have your studies ‘Yes, yes, I do’, you have your family. So, just very normal. And cool, but I wasn’t expecting that”—Participant 3
	Engaging in meaningful activities	“I need to occupy my mind”—Participant 21
		“When I pick up a book, I escape reality and enter a new one”—Participant 1

**Table 3:** Photovoice sessions (16 sessions).

Measures	Mean	Median	SD	
Participants’ attendance sessions	73.81%	79.16%	25.29%	
Photographs taken by each participant	6.5	5	6.20	
Satisfaction survey of each Photovoice session (52 answers)				
Quantitative part	Mean	Median	SD	Range (0 (low)–10 (high))
1. Degree of functioning of the activity	9.48	10	0.80	0–10
2. Timetable and duration of the activity	9.15	10	1.19	0–10
3. Overall level of satisfaction	9.48	10	0.92	0–10
Qualitative part	Overarching themes			Quotes
4. Strengths	Social interaction and support; empathy and sharing emotions; and learning and discovery			“Being able to listen to the points of view of other participants and share opinions”, “The empathy among attendees”, “It ended up being a very pleasant and friendly group”, “I really enjoy it when we get together as a group and exchange impressions, discovering things and places…. That’s what I think happened today, and it’s something I truly enjoyed”
5. Weaknesses	Dynamics and structure of the session; environmental comfort; and attendance and participation			“At the beginning of the activity, there were more people, and later there were fewer of us”, “We can almost never all be present”, “Going deeper”, “It felt short to me”, “Very hot in the classroom. A bit long in duration today”
6. Improvement actions	Group duration and structure, and environmental comfort (physical conditions)			“Do it in different places”, “Go outside to the street”, “Take more external material”, “More interesting questions”, “That the session was not excessively long”.

SD: Standard deviation.

**Table 4:** Comparison of standardized questionnaires pre- and post-intervention (*n* = 17).

	Pre-Intervention			Post-Intervention			Test		
Variable (Range)	Mean	Median	SD ^a^	Mean	Median	SD	T ^b^	Z ^c^	*p*-Value ^d^
Mental health recovery									
QPR (15–60)	37.53	38	16.1	36.29	37	12.34	0.54		*p* = 0.593
RAS–DS (38–152)	111.18	113	19.69	116.71	118	17.82	1.525		*p* = 0.147
Doing things I value (6–24)	19.35	20	3.48	20	21	3.58		0.79	*p* = 0.425
Looking forward (18–72)	50.35	51	11.83	52.06	50	8.79	0.84		*p* = 0.413
Managing my illness (7–28)	20.94	20	4.05	21.47	21	3.59	0.62		*p* = 0.540
Connecting and belonging (7–28)	20.53	21	3.22	21.94	22	3.21	2.51		*p* = 0.023
Quality of life and psychological well-being									
EQ-5D (5–15)	7.71	7	2.25	7.88	8	2.14	0.41		*p* = 0.687
EQ-5D scale (0–100)	56.47	65	28.31	60.59	60	20.30	0.90		*p* = 0.379
PWB (39–234)	142.53	143	35.1	142.88	145	33.16	0.08		*p* = 0.935
Self–acceptance (6–36)	19.53	16	7.68	18.35	16	6.55		0.96	*p* = 0.334
Positive relations with others (6–36)	21	22	6.72	22.65	23	7.85	1.13		*p* = 0.276
Autonomy (8–48)	29.29	30	8.86	29.35	30	7.7	0.060		*p* = 0.953
Environmental mastery (6–36)	20.65	20	7.32	20.53	19	6.31	0.12		*p* = 0.907
Purpose in life (7–42)	33	35	6.47	33.24	35	7.4		0.25	*p* = 0.800
Personal growth (6–36)	19.06	17	9.06	18.76	16	7.78	2.57		*p* = 0.801
Self-stigma in mental health									
ISMI (29–116)	58.94	56	12.68	58	59	12.79	0.87		*p* = 0.395
Aligment (6–24)	13.29	14	4.07	13.24	13	3.64	0.09		*p* = 0.924
Assumption of stereotype or self-stigma (7–28)	11.47	11	2.91	11.18	11	3.2	0.62		*p* = 0.545
Perceived discrimiation or experience of discrimination (5–20)	10.47	10	3.2	10.76	11	2.92	0.67		*p* = 0.509
Social isolation (6–24)	11.76	11	4.17	11.76	13	4.22	0.00		*p* = 1.000
Resistance to stigma (5–20)	11.94	11	3.47	11.06	11	2.9	1.06		*p* = 0.304

^a^ SD: standard deviation. ^b^ t indicates the statistics from Student’s *t*-test. ^c^ Z refers to the statistics from the Wilcoxon test. ^d^ *p*-value for comparison between pre- and post-intervention according to the paired Student’s *t*-test or Wilcoxon test. QPR: questionnaire about the process of recovery. EQ-5D: European Quality of Life-5 Dimensions. RAS-DS: Recovery Assessment Scale—Domains and Stages. PWB: Psychological Well-being Scale. ISMI: Internalized Stigma of Mental Illness Inventory.

**Table 5:** Satisfaction survey of “Artistic Couples” participation (*n* = 30).

Quantitative Part	Mean	Median	SD	Range (0 (Low)–10 (High))
1. Degree of functioning of the activity	8.9	9	1.53	0–10
2. Timetable and duration of the activity	8.7	9	1.83	0–10
3. Overall level of satisfaction	9	9	1.01	0–10
Qualitative part	Overarching themes		Quotes	
4. Strengths	Social interaction and personal connection; emotional and therapeutic support; andlearning and personal development		“Share moments with a person”, “For a moment you feel free of everything that disturbs you”, “Exploring different ways of expression with art”, “To interact and gain new knowledge from other people”	
5. Weaknesses	Lack of time and organization;space and logistics limitations; and compatibility and communication issues		“I didn’t have the chance to see my partner as much as I would have liked”, “The displacement distance to the artist’s studio”, “We are two people with very different character and styles and sometimes we have not fully understood or connected”	
6. Improvement actions	Having more time; having a shared creative space; andorganizing more meetings between artists		“Having more time”, “Have a creative space to do the project”, “Diversify in different disciplines and touching them all to enrich you”, “I would love to keep painting more. I enjoy recreational activities; they relax me”.	

SD: Standard deviation.

**Table 6:** Patient-reported experience measures (PREMs) (*n* = 30).

Questions	Definitely Yes	Yes	No	Definitely No	Do Not Want to Answer
Did the main researcher explain things in a way that was easy to understand?	19	11	0	0	0
Did the main researcher treat you with courtesy and respect?	22	8	0	0	0
Did you feel satisfied with the research process you experienced?	21	9	0	0	0

## Data Availability

The data presented in this study are available on request from the corresponding author due to ethical restrictions related to participant confidentiality.

## Abbreviations

The following abbreviations are used in this manuscript:

PROMs	Patient-Reported Outcome Measures
QPR	Questionnaire about the process of recovery
RAS-DS	Recovery Assessment Scale—Domains and Stages
EQ-5D	European Quality of Life-5 Dimensions
PWB	Ryff Psychological Well-being Scale
ISMI	Internalized Stigma of Mental Illness Inventory
PREMs	Patient-reported experience measures
